# Omega-3 fatty acid decreases oleic acid by decreasing SCD-1 expression in the liver and kidney of a cyclosporine-induced nephropathy rat model

**DOI:** 10.1080/0886022X.2019.1591996

**Published:** 2019-04-04

**Authors:** Su Mi Lee, Mi Hwa Lee, Young Ki Son, Seong Eun Kim, Yongsoon Park, Seo Hee Rha, Won Suk An

**Affiliations:** a Department of Internal Medicine, Dong-A University, Busan, Republic of Korea;; b Department of Anatomy and Cell Biology and Mitochondria Hub Regulation Center, Dong-A University, Busan, Republic of Korea;; c Department of Food and Nutrition, Hanyang University, Seoul, Republic of Korea;; d Department of Pathology, Dong-A University, Busan, Republic of Korea

**Keywords:** Cyclosporine, fatty acid, kidney, liver, omega-3, oleic acid

## Abstract

**Aim:** Stearoyl-CoA desaturase (SCD)-1 and elongase-6 (Elovl-6) are associated with fatty acid (FA) synthesis. We evaluated the effect of omega-3 FA on erythrocyte membrane FA contents through SCD-1 and Elovl-6 expression in the liver and kidney of a cyclosporine (CsA)-induced rat model.

**Methods:** Male Sprague Dawley rats were divided into control, CsA, and CsA treated with omega-3 FA groups. We measured SCD-1 and Elovl-6 expression levels via western blot and immunohistochemistry analysis.

**Results:** Erythrocyte membrane oleic acid content was lower in the CsA with omega-3 FA group compared to the CsA group. Compared to the control group, CsA-induced rats showed elevated SCD-1 expression in the kidney and liver, which omega-3 FA treatment reversed. Elovl-6 expression was increased in the liver, but decreased in the kidney in CsA group compared to control, which omega-3 FA treatment also reversed.

**Conclusions:** Omega-3 FA supplementation decreased erythrocyte membrane oleic acid content by modulating SCD-1 and Elovl-6 expression in the kidney and liver of CsA-induced rats.

## Introduction

Dyslipidemia is common in patients with chronic kidney disease (CKD) [1]. While kidney disease represented by chronic inflammatory conditions leads to alterations in lipid metabolism [2,3], lipid dysmetabolism is accompanied by progression of kidney disease [4,5]. The interaction between kidney dysfunction and lipid dysmetabolism produces a synergistic effect. Cyclosporine (CsA), an inhibitor of calcineurin, is used as an immunosuppressant, but long-term use of CsA can induce dyslipidemia as well as progressive renal disease [6,7].

Desaturases and elongases play a central role in synthesizing long chain monounsaturated fatty acid (MUFA) such as palmitoleic acid (16:1n-7) or oleic acid (18:1n-9) [8]. The elongase-6 (Elovl-6), a membrane-bound enzyme, plays key role by converting palmitic acid (16:0) to stearic acid (18:0) and is a condensing enzyme that initially determines the rate of FA elongation [9,10]. Stearoyl-CoA desaturase (SCD) is an endoplasmic reticular enzyme in the cell, biosynthesize MUFA from saturated fatty acid (SFA) and effects on key physiological variables especially in metabolism and atherosclerosis [9,11–14]. Mouse models with SCD-1 deficiency exhibited impaired synthesis of various kinds of lipids such as triglycerides (TG) and cholesterol esters [15,16]. The liver in particular plays an important role in these metabolic activities. Despite abundant studies regarding the regulation of elongases and desaturases in fatty acid (FA) metabolism in the liver, little is known about this subject in the kidney.

Omega-3 FA supplementation showed a definite decrease in erythrocyte membrane oleic acid content in several studies [17,18]. However, the mechanism for this effect remains unclear. The present study aimed to investigate the effect of omega-3 FA on the FA contents of erythrocyte membrane, and the SCD-1 and Elovl-6 expression in the kidney and liver of a CsA-induced rat model.

## Methods

### Animals and experimental design

Male Sprague Dawley rats, initially weighing 180–200 g, were housed in cages with temperature- and light-controlled environments. The animals were freely fed a low-salt diet (0.05% sodium; Teklad Premier, Madison, WI) and tap water. CsA (Chong Kun Dang, Seoul, Korea) was diluted with saline to a final concentration of 15 mg/mL. We used low-salt diet because salt depletion diet is known to enhance CsA-induced nephrotoxicity in the rats [19]. We used Omacor R (Kuhnil Pharm., Seoul, Korea) composed with eicosapentaenoic acid (EPA) 460.0 mg and docosahexaenoic acid (DHA) 380.0 mg for omega-3 FA treatment.

All animal procedures were approved by Dong-A University’s Institutional Animal Care Committee (DIACUC-14-29) and were conducted in conformity with the Public Health Service Policy on Human Care and Use of Laboratory Animals. The rats were divided into three groups: group 1 (*n* = 8) was administered saline (1 mL/(kg⋅day)) via subcutaneous injection for 4 weeks, group 2 (*n* = 8) was administered CsA (15 mg/(kg⋅day)) via subcutaneous injection for 4 weeks, and group 3 (*n* = 8) was administered CsA and omega-3 FA (300 mg/(kg⋅day)) via gastric gavage for 4 weeks. All the rats survived this phase of the experiment. The selected CsA dosage and administration route previously proved to effectively induce renal injury [20]. The omega-3 FA treatment (dosage and administration route) was also decided through another previous study [21]. The rats were fed evenly and their body weights were monitored daily. After the 4-week treatments, the rats were killed using diethyl ether anesthesia and blood samples were collected from the heart. Blood urea nitrogen (BUN) and serum creatinine (sCr) levels were evaluated via an automatic analyzer (Roche, Basel, Germany). Whole blood CsA levels were determined via monoclonal radioimmunoassay (Incstar, Stillwater, MN). Total cholesterol (TC) and TG levels were measured using colorimetric test kits (Asan Pharmaceutical Co., Seoul, Korea) purchased from specified manufacturers.

### Gas chromatography analysis

FA contents in erythrocyte membrane, kidney, and liver were analyzed via gas chromatography (Shimadzu GC-2010AF, Shimadzu Scientific Instrument, Kyoto, Japan). Kidney and liver tissues (100 mg) were homogenized with 5 mL of chloroform:methanol:distilled water (2:2:1 v/v). Fats extracted from kidney and liver tissues were separated on thin layer chromatography (Silica gel G, Analtech, Newark, DE) using hexane:ether:acetic acid (40:10:1, v/v). Isolated erythrocytes, kidney, and liver phospholipid were methylated by the addition of boron trifluoride (BF3) methanol-benzene for 10 min at 100 °C. FA methyl esters were analyzed via gas chromatography with a 100 m SP2560 capillary column (Supelco, Bellefonte, PA). FA was identified after comparing with known standards (GLC-727; Nu-Chek Prep, Elysian, MN). The omega-3 index is a measure of EPA and DHA [22]. FA contents were expressed as weight percentages. Activities of elongase and SCD-1 are estimated as the ratio of stearic acid/palmitic acid [23] and palmitoleic acid/palmitic acid [24].

### Histopathologic evaluation

On the day of sacrifice, kidney and liver tissues were excised, washed with heparinized saline, fixed in periodate-lysine-paraformaldehyde solution, and embedded in wax. After deparaffinization, 4-μm thick sections were processed and stained with the periodic acid-Schiff. Kidney lesions such as tubular atrophy, inflammatory cellular infiltrates, and interstitial fibrosis were viewed using an Aperio Scan Scope slide scanner (Aperio Technologies, Vista, CA). For measuring vacuolization of tubular cells, a computer-assisted quantitative analysis (CaseViewer 2.2, 3DHISTECH Ltd, Budapest, Hungary) was performed. In each specimen, five randomly selected non-overlapping high-power fields were acquired by using ×20-objective lens and then analyzed by using the Image-Pro Plus software. The quantitative renal tubular vacuolization areas were calculated by dividing with the total stained areas in each slide.

### Western blot analysis

Kidney and liver tissues were homogenized in lysis buffer containing 300 mM NaCl, 50 mM Tris–HCl, 0.5% Triton X-100, and protease-inhibitor cocktail (pH 7.6). The incubation was performed at 4 °C for 30 min. After centrifugation at 14,000 rpm for 20 min at 4 °C, protein concentrations of the lysates were determined with the Bradford protein assay reagent (Bio-Rad, Hercules, CA). The proteins (40 μg) were loaded into 7.5–15% SDS/PAGE gels and transferred onto a nitrocellulose membrane (Amersham Pharmacia Biotech, Piscataway, NJ). The membranes were incubated overnight in blocking buffer at 4 °C with each antibody. Antibodies against sterol regulatory element-binding protein (SREBP)-1 and liver X receptor (LXR) were obtained from Santa Cruz Biotechnology (Santa Cruz, CA). Antibodies against SCD-1 and Elovl-6 were purchased from Abcam (Cambridge, MA). Antibodies against β-actin were obtained from Sigma (St. Louis, MO). The membranes were subsequently incubated with horseradish peroxidase-conjugated secondary antibody for 60 min at room temperature. Immunostaining with antibodies was performed using the Super Signal West Pico Chemiluminescent Substrate (Thermo Scientific, Hudson, NH) and detected with the LAS-3000 Plus (Fuji Photo Film, Tokyo, Japan). The samples were quantified and normalized against the β-actin control using ImageJ version 1.48.

### Immunohistochemical staining

To perform immunohistochemical (IHC) staining for SCD-1 and Elovl-6, kidney sections were transferred into a Dako Target Retrieval Solution (pH 9.0; *DAKO*, Carpinteria, CA). The slides were microwaved at medium power for 15 min to retrieve antigens. To block endogenous peroxidase activity, the tissue sections were treated with 3% H_2_O_2_ in distilled water for 10 min, and further incubated with 5% normal goat serum at room temperature for 30 min and Protein Block Serum-Free solution (*DAKO*, Carpinteria, CA) for 10 min. The slides were incubated overnight with anti-SCD-1 and anti-Elovl-6 antibodies at 4 °C, and further incubated with secondary antibodies for 1 h at 37 °C. The slides were then incubated in 3,3-diaminobenzidine + H_2_O_2_ substrate and hematoxylin. The negative controls were stained under identical conditions, but with a buffer solution instead of the primary antibody. The results were viewed using an Aperio Scan Scope slide scanner.

### Statistical analysis

The data are presented as mean ± SD or frequency (count and percentage). The subjects’ characteristics were analyzed using Mann–Whitney’s *U* test for continuous variables, Chi-squared test for categorical variables, and the Kruskal–Wallis test for continuous variables within three groups. All the analyses were performed using the SPSS version 18.0 software (SPSS Inc., Chicago, IL). All analyses were considered significant when a *p* value of <.05 was identified.

## Results

### Baseline characteristics

The biochemical characteristics are summarized in Table 1. At 4 weeks, BUN and sCr levels were significantly higher in the CsA-treated and CsA with omega-3 FA-treated groups when compared to the control group. At the end of study, CsA levels were >2000 ng/mL in the CsA-treated group and CsA with omega-3 FA-treated group, without exhibiting any significant difference in either group. The TC and TG levels were also significantly elevated in the CsA group when compared to the control group; however, the data showed a decreasing trend after omega-3 FA treatment.

**Table 1.** Biochemical tests in each group.

	Control (*n* = 8)	Cyclosporine (*n* = 8)	Cyclosporine with omega-3 FA (*n* = 8)	*p* Value
Final body weight (g)	432.5 ± 7.4	338.3 ± 5.7	340.3 ± 12.0	
Blood urea nitrogen (mg/dL)	17.7 ± 1.5	62.9 ± 11.3*	51.6 ± 8.2*	<.001
Creatinine (mg/dL)	0.40 ± 0.04	0.64 ± 0.09*	0.60 ± 0.09*	.004
Cyclosporine		2542.5 ± 623.8	2418.1 ± 683.5	.405
Total cholesterol (mg/dL)	61.9 ± 7.3	109.2 ± 10.5*	90.3 ± 17.3*	.008
Triglyceride (mg/dL)	134.0 ± 27.2	354.5 ± 161.8*	291.5 ± 83.5*	.027

Data are expressed as means ± SD.

*p< .05, compared to control group.

### Comparisons of fatty acid contents in erythrocyte membrane

The erythrocyte membrane contents of SFA, stearic acid, and lignoceric acid contents were observed to be significantly decreased, whereas myristic and palmitic acid contents were significantly increased in the CsA-treated group when compared to the control group (Table 2). However, omega-3 FA supplementation reversed the total SFA content and markedly increased palmitic acid content. Omega-6 and arachidonic acid (AA) contents were lower in the omega-3 FA-supplemented CsA group than in the CsA group. Omega-3 index (EPA and DHA contents) is a new risk factor for death from coronary heart disease [22]. The EPA, DHA, and omega-3 index significantly increased in the omega-3 FA supplemented CsA group when compared to the CsA group. Additionally, the ratio of AA to EPA and omega-6 to omega-3 also significantly decreased in the omega-3 FA-supplemented CsA group when compared to the CsA group. Finally, the oleic acid content in erythrocyte membrane significantly increased in the CsA group when compared to the control group, which was significantly decreased by omega-3 FA supplementation.

**Table 2.** Comparisons of erythrocyte membrane fatty acid contents.

	Control (*n* = 8)	Cyclosporine (*n* = 8)	Cyclosporine with omega-3 FA (*n* = 8)	*p* Value
Saturated	42.8 ± 0.6	40.8 ± 1.4*	42.8 ± 0.7**	.009
Myristic	0.27 ± 0.04	0.41 ± 0.07*	0.36 ± 0.10	.020
Palmitic	25.1 ± 1.3	26.9 ± 1.5*	29.0 ± 0.4*^,^**	.001
Stearic	17.0 ± 1.7	13.3 ± 0.9*	13.3 ± 0.7*	.003
Lignoceric	0.47 ± 0.05	0.24 ± 0.08*	0.21 ± 0.04*	.001
Monounsaturated	11.6 ± 0.8	14.9 ± 1.1*	13.5 ± 1.0*	.001
Palmitoleic	0.41 ± 0.19	1.4 ± 0.3*	1.0 ± 0.5*	.001
Oleic	10.7 ± 0.6	13.1 ± 0.7*	12.0 ± 0.5*^,^**	.001
Polyunsaturated	44.7 ± 0.6	43.2 ± 0.8*	42.7 ± 0.9*	.003
Omega-6	38.6 ± 1.6	40.7 ± 0.7*	36.0 ± 1.0*^,^**	.001
Linoleic	9.3 ± 0.8	11.0 ± 1.1*	13.2 ± 1.4*^,^**	.002
AA	25.9 ± 1.5	24.2 ± 1.1*	19.8 ± 1.2*^,^**	.001
Omega-3	6.2 ± 1.8	2.5 ± 0.1*	6.7 ± 0.8**	.003
Alpha-linolenic	0.06 ± 0.02	0.09 ± 0.05	0.10 ± 0.05	.442
EPA	0.48 ± 0.30	0.09 ± 0.04*	0.81 ± 0.15**	.001
DHA	3.6 ± 0.7	1.7 ± 0.9*	3.8 ± 0.5**	.003
Omega-3 index	4.1 ± 0.9	1.8 ± 0.1*	4.6 ± 0.6**	.002
AA/EPA	91.7 ± 73.7	370.8 ± 270.1*	25.3 ± 5.0*^,^**	<.001
Omega-6/omega-3	6.9 ± 2.4	16.6 ± 0.7*	5.5 ± 0.7**	.002

AA: arachidonic acid; EPA: eicosapentaenoic acid; DHA: docosahexaenoic acid; SCD: stearoyl-CoA desaturase.

Data are expressed as means ± SD.

*p < .05, compared to control group.

**p < .05, compared to cyclosporine group.

### Elongase activity based on phospholipid ratio in kidney and liver

While the palmitic acid content in the CsA was higher in kidney and lower in liver than the control group, the stearic acid content in the CsA group was lower in kidney and higher in liver than the control group (Table 3). Additionally, the elongase activity in kidney significantly decreased in the CsA group, and that in liver changed in the opposite direction.

**Table 3.** Comparisons of phospholipid contents related with elongase and SCD-1 activities in kidney and liver.

	Control (*n* = 8)	Cyclosporine (*n* = 8)	Cyclosporine with omega-3 FA (*n* = 8)	*p* Value
Kidney				
Palmitic	20.7 ± 0.8	21.7 ± 0.9*	22.1 ± 0.4*	.005
Stearic	23.6 ± 1.6	21.5 ± 0.9*	21.2 ± 0.7*	.015
Elongase (18:0/16:0)	1.14 ± 0.10	0.99 ± 0.03*	0.96 ± 0.03*	.001
SCD-1 (16:1n7/16:0)	0.02 ± 0.00	0.04 ± 0.00*	0.04 ± 0.01*	.001
Liver				
Palmitic	17.9 ± 1.2	17.1 ± 0.5	18.5 ± 0.5**	.015
Stearic	28.9 ± 1.2	31.7 ± 0.5*	28.5 ± 0.4**	.002
Elongase (18:0/16:0)	1.62 ± 0.14	1.86 ± 0.07*	1.54 ± 0.05**	.006
SCD-1 (16:1n7/16:0)	0.04 ± 0.01	0.04 ± 0.00	0.03 ± 0.00^*,**^	.012

Data are expressed as means ± SD.

*p < .05, compared to control group.

**p < .05, compared to cyclosporine group.

### Renal pathology

**Figure 1.**
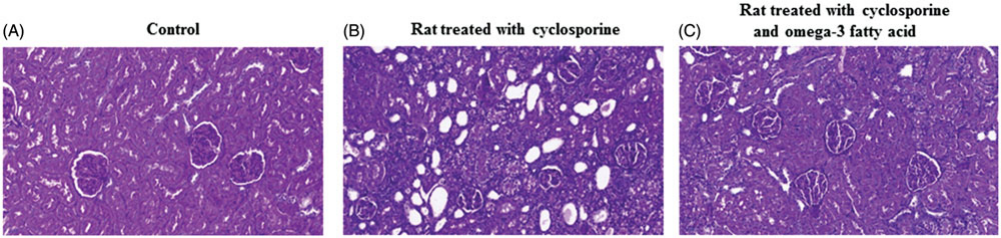
Kidney morphological change among three groups. (A) Periodic acid-Schiff-stained specimens from the control. (B) The cyclosporine induced rat revealed vacuolization of tubular cells, tubular atrophy and interstitial fibrosis. (C) The cyclosporine induced rat treated with omega-3 FA had less tubulointerstitial changes than the cyclosporine induced rat.

As shown in Figure 1, periodic acid-Schiff-stained specimens from the CsA-treated group revealed vacuolization of tubular cells, tubular atrophy, and interstitial fibrosis (Figure 1(A,B)). The omega-3 FA-supplemented CsA group exhibited lesser tubulointerstitial changes than the group treated with CsA alone (Figure 1(C)).

**Figure 2.**
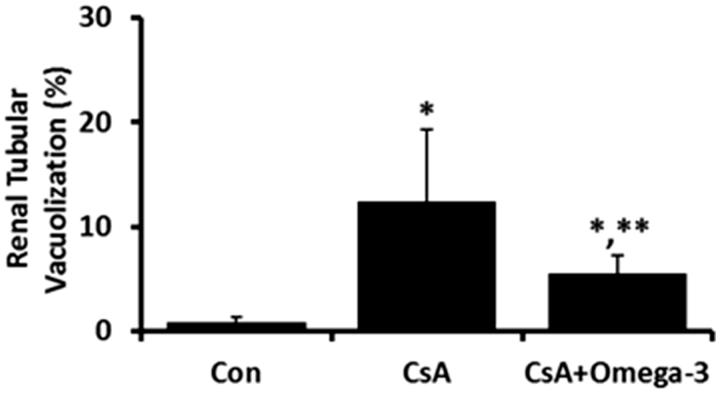
Expression of tubular vacuolization in kidney. The tubular vacuolization areas in kidney were increased in the CsA-induced rats, but were decreased by omega-3 FA supplementation. *p < .05, compared to control group, **p < .05, compared to cyclosporine group.

The tubular vacuolization areas in kidney were increased in the CsA-treated group, but were decreased by omega-3 FA supplementation (Figure 2).

#### SCD-1 and Elovl-6 in the kidney and liver

**Figure 3.**
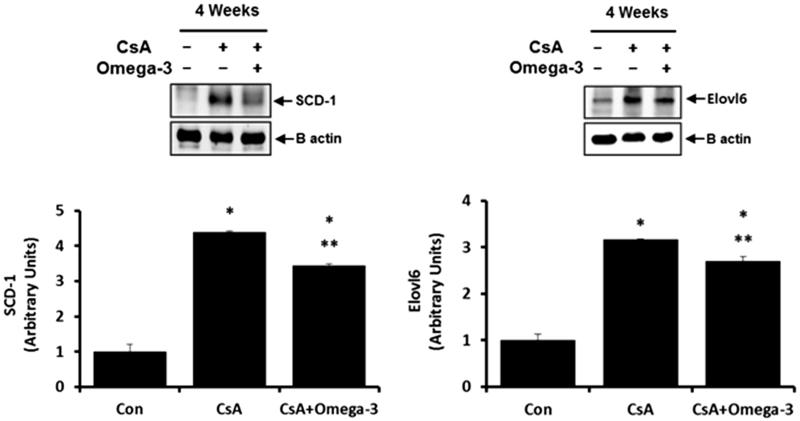
SCD-1 and Elovl-6 expression in liver of cyclosporine induced rat. Compared with the control group, SCD-1 and Elovl-6 expressions were increased in liver of cyclosporine group and omega-3 FA reversed these effects in western blotting. *p < .05, compared to control group, **p < .05, compared to cyclosporine group.

**Figure 4.**
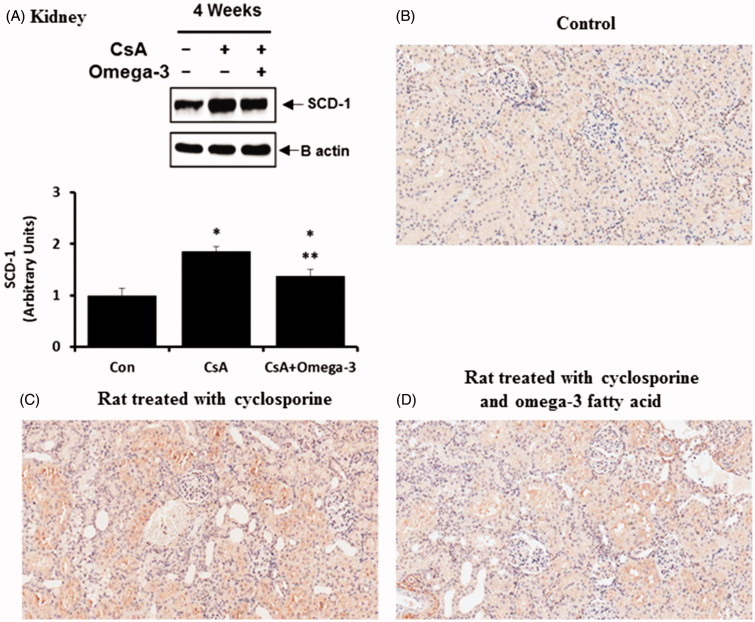
SCD-1 expression in kidney of cyclosporine induced rat. (A) Compared with the control group, SCD-1 expression was increased in kidney of cyclosporine group and omega-3 FA attenuated this up-regulation in western blotting of kidney. (B) Immunohistochemically stained specimens from the control. SCD-1 was mainly expressed in tubules of normal control. (C) SCD-1 expression was markedly increased in the kidney of cyclosporine induced rat. (D) SCD-1 expression was decreased by omega-3 FA supplementation in the kidney of cyclosporine induced rat. *p < .05, compared to control group, **p < .05, compared to cyclosporine group.

**Figure 5.**
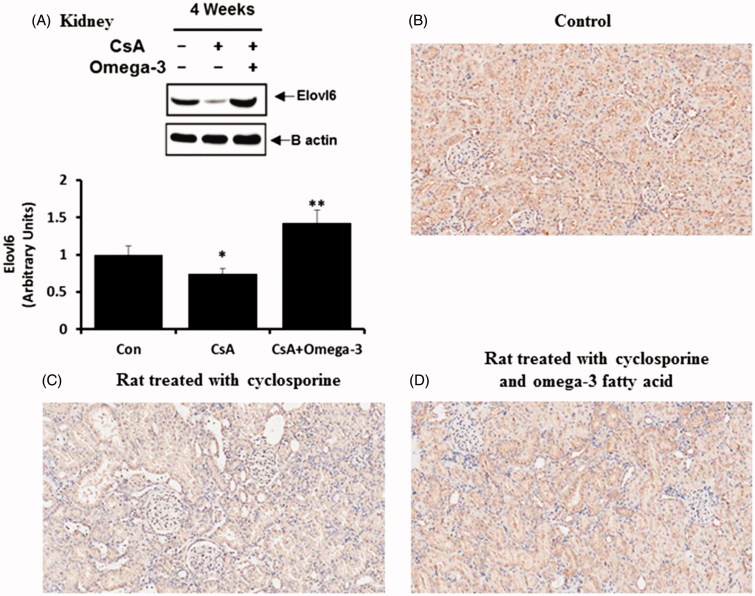
Elovl-6 expression in kidney of cyclosporine induced rat. (A) Compared with the control group, Elovl-6 expression was decreased in kidney of cyclosporine group and omega-3 FA up-regulated Elovl-6 expression in western blotting of kidney. (B) Immunohistochemically stained specimens from the control. Elovl-6 was markedly expressed in tubules of normal control. (C) Elovl-6 expression was decreased in the kidney of cyclosporine induced rat. (D) Elovl-6 expression was reversed by omega-3 FA supplementation in the kidney of cyclosporine induced rat. *p < .05, compared to control group, **p < .05, compared to cyclosporine group.

As demonstrated by the western blotting analysis, SCD-1 and Elovl-6 expression levels were increased in the liver of the CsA-treated group when compared to the control group, the effects of which were reversed by omega-3 FA (Figure 3). The western blots of the kidney samples from the CsA group showed a significant elevation in SCD-1 expression when compared to the control group (Figure 4(A)). Omega-3 FA supplementation attenuated this upregulation in the kidney. Renal SCD-1 expression was also identified by performing an IHC stain. SCD-1 was mainly expressed in the tubules of the normal control group (Figure 4(B)) and its expression was markedly increased in the kidney of the CsA-induced rats, but was decreased by omega-3 FA supplementation (Figure 4(C,D)). Western blot analysis of the kidney samples also demonstrated that the CsA group showed significantly decreased Elovl-6 expression, which omega-3 FA upregulated (Figure 5(A)). Elovl-6 was markedly expressed in the tubules of the normal control group after IHC staining (Figure 5(B)). Its expression level was decreased in the kidney of CsA-induced rats, which was reversed by omega-3 FA supplementation (Figure 5(C,D)).

#### SREBP-1 and LXR in the kidney

**Figure 6.**
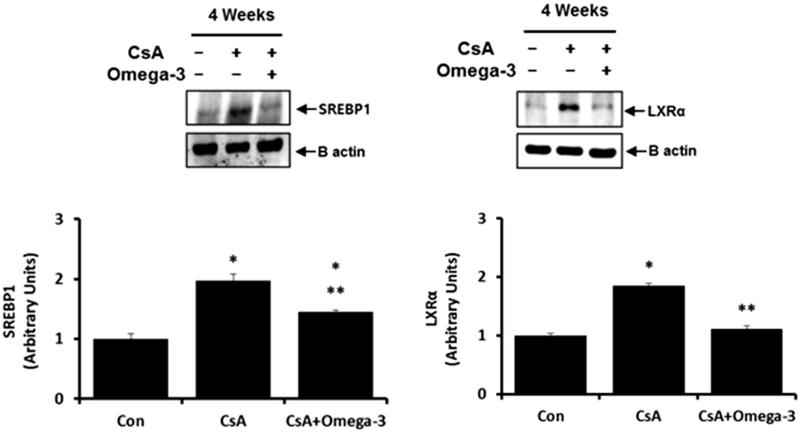
SREBP-1 and LXR expression in kidney of cyclosporine induced rat. Compared with the control group, SREBP-1 and LXR expressions were up-regulated in kidney of cyclosporine group, which was down-regulated with omega-3 FA supplementation in western blotting. *p < .05, compared to control group, **p < .05, compared to cyclosporine group.

Figure 6 shows the quantitative analysis of SREBP-1 and LXR. SREBP-1 and LXR expressions were observed to be upregulated in CsA-induced rat kidney when compared to control, which was downregulated with omega-3 FA supplementation.

## Discussion

In this study, we found that omega-3 FA supplementation decreased the erythrocyte membrane oleic acid content in a CsA-induced nephropathy rat model showing higher oleic acid content compared to normal control. In addition, SCD-1 expression increased in both the kidney and liver of the CsA-induced nephropathy rat model, which was found to be reversed by omega-3 FA supplementation. SCD-1 is a 37-kDa transmembrane protein containing four domains [25] that is required for the synthesis of MUFA, including oleic acid, from SFA [26,27]. While SCD-1 is expressed in adipose and eyelid tissue under normal dietary conditions [16], it is highly expressed in the liver and heart under a high-carbohydrate diet [28]. Higher SCD‐1 expression was reported under increased SFA and MUFA deposits in the liver of male Swiss mice fed a high-carbohydrate diet [23]. Therefore, increased erythrocyte membrane oleic acid contents and higher SCD‐1 expressions of kidney and liver in a CsA-induced nephropathy rat model reflect that CsA-induced nephropathy induces metabolic disorder like feeding high-carbohydrate diet. In previous studies, reduced expression of SCD-1 was also found in the liver of mice model supplemented with DHA and EPA-enriched phospholipid diet [29,30]. On the basis of this study and several previous studies, reduced erythrocyte membrane oleic acid contents caused by omega-3 FA supplementation can be explained by the inhibition of SCD-1 activity in the liver. Furthermore, the effect of omega-3 FA on SCD-1 suppression was also found in the kidney of this study. To our knowledge, this is the first report that showed up-regulation of SCD-1 in the status of renal injury and omega-3 FA-mediated SCD-1 suppression in the kidney. It is of note that kidney is also important organ modulating FA metabolism.

Previous study showed that both SCD-1 and Elovl-6 activities were up-regulated and the oleic acid level was elevated in obese Zucker rats [31]. The oleic acid levels were decreased in Elovl-6-/- mice model [9]. This suggests that SCD-1 and Elovl-6 may be simultaneously activated in the liver and Elovl-6 expression may be related with oleic acid contents. Our study also showed that SCD-1 and Elovl-6 expressions were increased in the liver of the CsA-induced nephropathy rat model and their enzyme expressions and oleic acid contents were decreased after omega-3 FA supplementation. However, Elovl-6 expression in the kidney was observed to contradict its expression in the liver of rats that underwent the CsA and omega-3 FA treatment. Additionally, stearic acid contents were expressed in opposite directions in liver and kidney of CsA group. Elovl-6 prefers SFA and MUFA as substrates [29,31] and is highly expressed in the liver and white adipose tissue of mice in the re-fed state after fasting [32]. In terms of *de novo* MUFA biosynthesis, stearic acid is synthesized by Elovl-6 from palmitic acid. A decrease in Elovl-6 in the kidney of CsA-induced nephropathy rat may have led to a decrease in the conversion from palmitic acid to stearic acid. Similarly, an increase in stearic acid in the liver, unlike in the kidney, may be explained by an increase in Elovl-6 expression in the liver of CsA-induced nephropathy rat. Elongase activity based on phospholipids ratio of liver and kidney was similar with Elovl-6 expressions of liver and kidney in this study. The exact mechanism cannot be explained through this study, but it can be deduced that along with the liver, the kidney also partly contributes to lipogenic metabolism. Elevation of Elvol-6 expression in the kidney after omega-3 FA administration may be a compensatory action to the liver trying to convert palmitic acid to stearic acid. Omega-3 FA may act as a modulator in the complex process of FA elongation and desaturation. Further studies are necessary to support this assumption and mechanism.

It has been reported that several transcription factors such as SREBP-1 and LXR regulated desaturases and elongases during lipogenesis [32]. SREBP is a membrane-bound transcription factor that belongs to the basic-helix-loop-helix leucine zipper family [33] and has been established as a regulator for the biosynthesis of both, cholesterol and FA. Increased SREBP-1 and LXR activities upregulate SCD-1 and Elovl-6 gene expression, which increases the conversion of palmitic acid and stearic acid to palmitoleic acid and oleic acid [32,34–36]. It is well-known that liver is an important place for this activity. In our study, renal SREBP-1, LXR, and SCD-1 expression levels increased, but Elovl-6 expression was decreased in the CsA-induced nephropathy rat model. Based on our study, it can be deduced that the kidney also contributes to lipid metabolism like liver. However, it is difficult to explain why Elovl-6 expression was decreased in the kidney. Also, the interaction between Elovl-6 and SCD-1 in kidney is not clear. We assume that SREBP-1 and LXR predominantly regulate SCD-1 expression instead of Elovl-6 in the kidney.

Previous studies showed that omega-3 FA supplementation improved lipid profiles and interstitial fibrosis in human and CKD rat model [37,38]. Although BUN and sCr levels were not changed between CsA-treated group and CsA with omega-3 FA-treated group, pathologic changes such as vacuolization of tubular cells, tubular atrophy, and interstitial fibrosis were found in CsA-treated group. These findings were attenuated by omega-3 FA supplementation in this study. Therefore, our study demonstrated that omega-3 FA was beneficial for CsA-induced lipid dysmetabolism and renal injury.

## Conclusions

Omega-3 FA supplementation decreased the erythrocyte membrane oleic acid content by modulating SCD-1 and Elovl-6 expression in the kidney and liver of CsA induced nephropathy rats. Further studies are needed to elucidate the crosstalk between the liver and kidney with respect to SCD-1 and Elovl-6 activities in the CKD rat model.
